# Heterologous overproduction of oviedomycin by refactoring biosynthetic gene cluster and metabolic engineering of host strain *Streptomyces coelicolor*

**DOI:** 10.1186/s12934-023-02218-8

**Published:** 2023-10-14

**Authors:** Boncheol Gu, Duck Gyun Kim, Do-Kyung Kim, Minji Kim, Hyun Uk Kim, Min-Kyu Oh

**Affiliations:** 1 Department of Chemical & Biological Engineering, Korea University, Seoul, 02841 Republic of Korea; 2 Department of Chemical and Biomolecular Engineering (BK21 four), Korea Advanced Institute of Science and Technology (KAIST), Daejeon, 34141 Republic of Korea

**Keywords:** Metabolic engineering, Oviedomycin, Refactoring, Heterologous expression, Genome-scale metabolic model

## Abstract

**Background:**

Oviedomycin is one among several polyketides known for their potential as anticancer agents. The biosynthetic gene cluster (BGC) for oviedomycin is primarily found in *Streptomyces antibioticus*. However, because this BGC is usually inactive under normal laboratory conditions, it is necessary to employ systematic metabolic engineering methods, such as heterologous expression, refactoring of BGCs, and optimization of precursor biosynthesis, to allow efficient production of these compounds.

**Results:**

Oviedomycin BGC was captured from the genome of *Streptomyces antibioticus* by a newly constructed plasmid, pCBA, and conjugated into the heterologous strain, *S. coelicolor* M1152. To increase the production of oviedomycin, clustered regularly interspaced short palindromic repeats/CRISPR-associated protein 9 (CRISPR/Cas9) system was utilized in an in vitro setting to refactor the native promoters within the *ovm* BGC. The target promoters of refactoring were selected based on examination of factors such as transcription levels and metabolite profiling. Furthermore, genome-scale metabolic simulation was applied to find overexpression targets that could enhance the biosynthesis of precursors or cofactors related to oviedomycin production. The combined approach led to a significant increase in oviedomycin production, reaching up to 670 mg/L, which is the highest titer reported to date. This demonstrates the potential of the approach undertaken in this study.

**Conclusions:**

The metabolic engineering approach used in this study led to the successful production of a valuable polyketide, oviedomycin, via BGC cloning, promoter refactoring, and gene manipulation of host metabolism aided by genome-scale metabolic simulation. This approach can be also useful for the efficient production of other secondary molecules encoded by ‘silent’ BGCs.

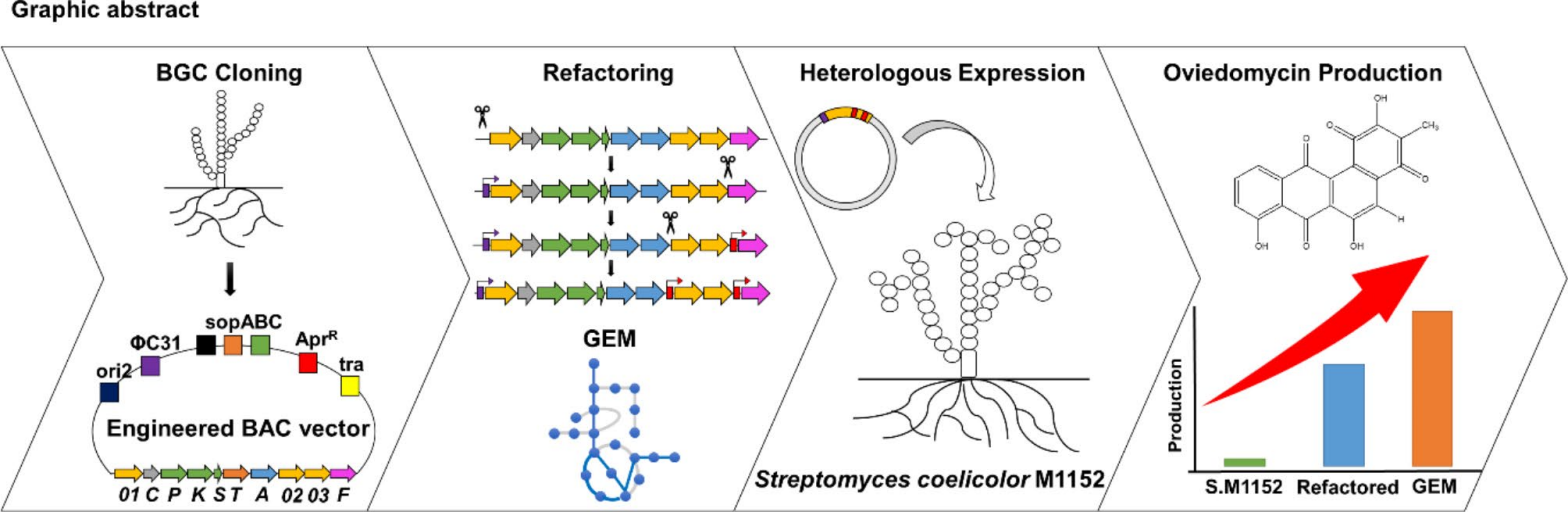

**Supplementary Information:**

The online version contains supplementary material available at 10.1186/s12934-023-02218-8.

## Background

Given the rapid growth of the global pharmaceutical sector, there is a growing demand for research focused on life-threatening diseases such as tumors and other substantial health risks. Consequently, it is imperative to investigate innovative drug candidates to align with industrial progress and effectively address these challenges [1]. Actinomycetes synthesize a diverse array of polyketides with potential pharmaceutical applications such as antivirals, antibiotics [2, 3], immunosuppressants [4, 5], and anticancer agents [6, 7]. One such polyketide is oviedomycin, which belongs to the type II angucycline polyketide group and is primarily found in *Streptomyces antibioticus*. Oviedomycin is a highly oxygenated type II polyketide derived from a synthesis process utilizing one acetyl-CoA and nine malonyl-CoA’s. Polyketides are produced through the collaboration of enzymes encoded in gene groups known as biosynthetic gene clusters (BGCs). In the *ovm* BGC, Type II polyketide synthase genes (*ovmPKSTAC*) encode enzymes for an angucycline structure comprising these precursors and the products of post-PKS tailored oxygenase genes (*ovm01, ovm02, and ovm03*) are involved in transforming it into the oxygenated structure characteristic of oviedomycin. It exhibits various biological activities, especially cytotoxic activities against human cancer cell lines, which sets it apart from other angucyclines and angucyclinones [8, 9].

Unfortunately, a significant portion of BGCs found in microorganisms remain transcriptionally inactive, commonly referred to as ‘silent’ [10, 11], indicating their lack of expression under normal laboratory conditions [12–16]. Various engineering techniques have been utilized to activate silent BGCs; however, inducing functional expression of many BGCs remains challenging [17]. To overcome this, heterologous expression has been proposed as a useful approach to aid in the synthesis of products encoded by silent BGCs [18, 19]. This approach offers a viable alternative by overcoming the challenges related to the cultivation and genetic manipulation of native strains.

Despite significant advancements in the heterologous expression of BGCs, this approach frequently falls short in terms of efficiency and productivity [20]. To enhance its efficiency, additional genetic modifications, such as BGC refactoring and host strain metabolic engineering, are necessary. Clustered regularly interspaced short palindromic repeats/CRISPR-associated protein 9 (CRISPR/Cas9) system has been effectively employed to manipulate the genome of *Streptomyces* strains [21]. Nonetheless, this approach is associated with challenges, including issues with the expression of the *Cas9* gene or single guide RNAs (sgRNAs), as well as potential detrimental effects associated with Cas9 [22, 23]. In vitro CRISPR/Cas9 strategies have been successful in addressing these issues and improving the production of secondary metabolites by refactoring BGCs [24]. Refactoring, which involves manipulating the genes that govern the pathway or redesigning specific genetic elements with better components, is the key process.

Metabolic engineering of host strains using genome-scale metabolic model (GEM) has been suggested as a promising strategy to improve the secondary metabolite production [25, 26]. The GEM is a computational model that can predict metabolic flux distributions associated with the precursors of secondary metabolites under certain conditions, rendering it a valuable tool in the systems metabolic engineering [27–29]. Using GEM, it is possible to identify specific target genes for deletion and/or overexpression to enhance the production of desired secondary metabolites.

In this study, we examined a combination of strategies to enhance the production of oviedomycin, including heterologous expression of the *ovm* BGC, refactoring of the BGC promoter, and genetic engineering of the host strain. Through combined engineering strategies, we could achieve the production of 670 mg/L oviedomycin, the highest titer reported to date. This study demonstrates the potential of combined engineering strategies to enhance the production of secondary metabolites. These findings suggest that this approach is promising for improving the production of various compounds and unlocking the potential of other bioactive molecules encoded within silent BGCs.

## Results

### Capture and expression of *ovm* BGC in the production host

To express *ovm* in a heterologous strain, a novel BGC cloning vector was designed to improve the efficiency of cloning long DNA fragments in *E. coli*. Previous studies have used pSET152 to capture secondary metabolite BGCs [30, 31]; however, we failed to obtain an *ovm*-cloned vector with pSET152. The inability to successfully clone the *ovm* BGC in *E. coli* utilizing a high copy number plasmid could be attributed to the extended length, elevated GC content, and recurring sequences of BGC, which might place a significant load on the host system, ultimately resulting in failure. Therefore, we designed a low-copy plasmid by integrating pSET152 with a bacterial artificial chromosome (BAC) vector, pCAP-BAC, [32] and generated a new plasmid, pCAP-BAC-Apr (pCBA) (Fig. S1a), using the essential components of both plasmids.

Next, linearized pCBA was created using the restriction enzymes: Swal and BamH1. Subsequently, the *ovm* BGC fragments generated by PCR were ligated to pCBA using Gibson assembly, resulting in the successful cloning of the *ovm* BGC. The resultant vector was named pCBAO. The pCBAO vector was then transformed into *E. coli* ET12567 (pUZ8002) and subsequently conjugated with *S. coelicolor* M1152, ultimately generating SCMO (Fig. S1b).

**Fig. 1:**
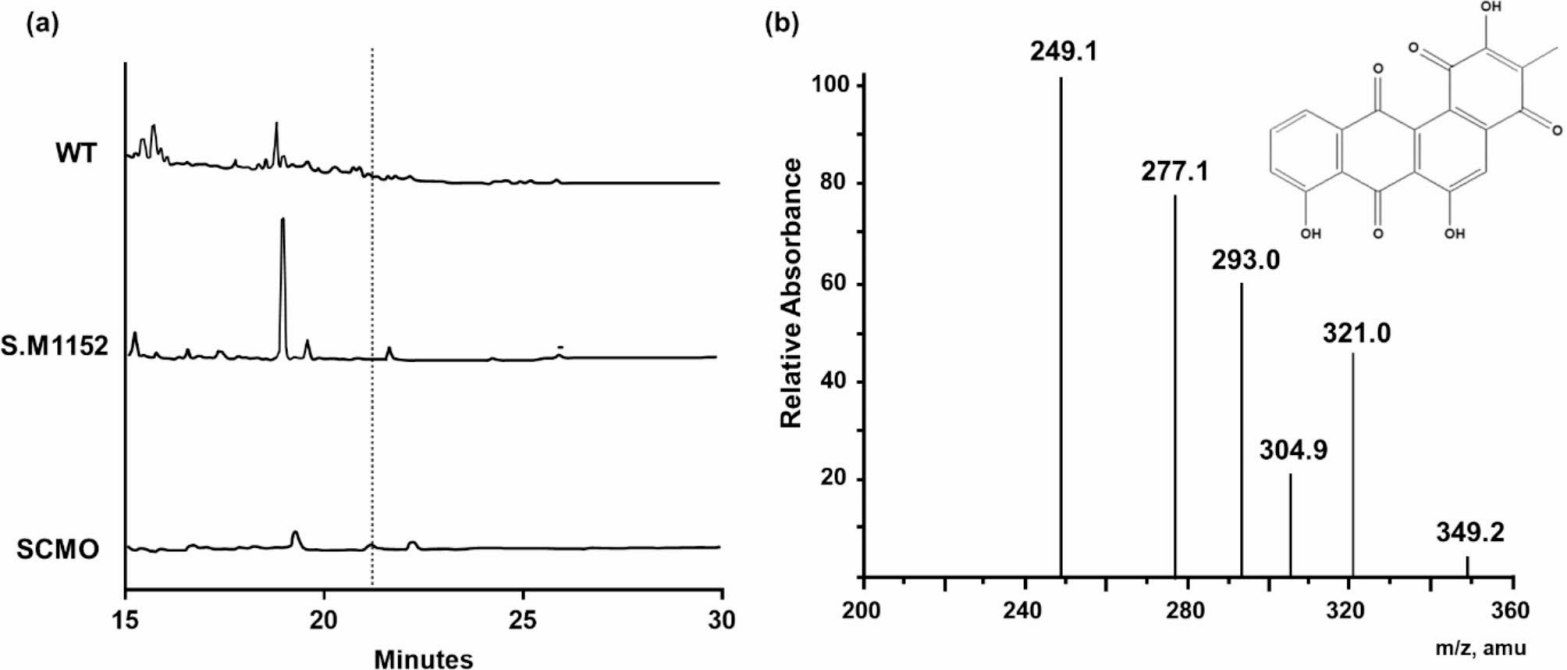
Analysis of oviedomycin production in native and heterologous strains. (**a**) HPLC chromatograms of cell extracts of *S. antibioticus* NRRL 3238 (WT), *S. coelicolor* M1152 (S. M1152), and SCMO were analyzed. Oviedomycin peak in the chromatogram is marked with a dotted line. (**b**) LC-MS/MS analysis of the oviedomycin peak in SCMO samples and the predicted structure

The production of oviedomycin by the heterologous strain SCMO was examined. As expected, neither the original source of *ovm* BGC, *S. antibioticus* NRRL 3238, nor *S. coelicolor* M1152 without *ovm* BGC, could produce oviedomycin, which was consistent with previous studies [9, 33] (Fig. 1a). SCMO produced only a small amount of oviedomycin, as observed in the HPLC chromatogram (Fig. 1a). Oviedomycin production was further confirmed by LC-MS/MS analysis (Fig. 1b) [33]. After cultivating SCMO in the production medium for seven days, the amount of oviedomycin produced was quantified as 1.24 mg/L.

### Refactoring *ovm* BGC via in vitro CRISPR/Cas9 to improve production yield

**Fig. 2:**
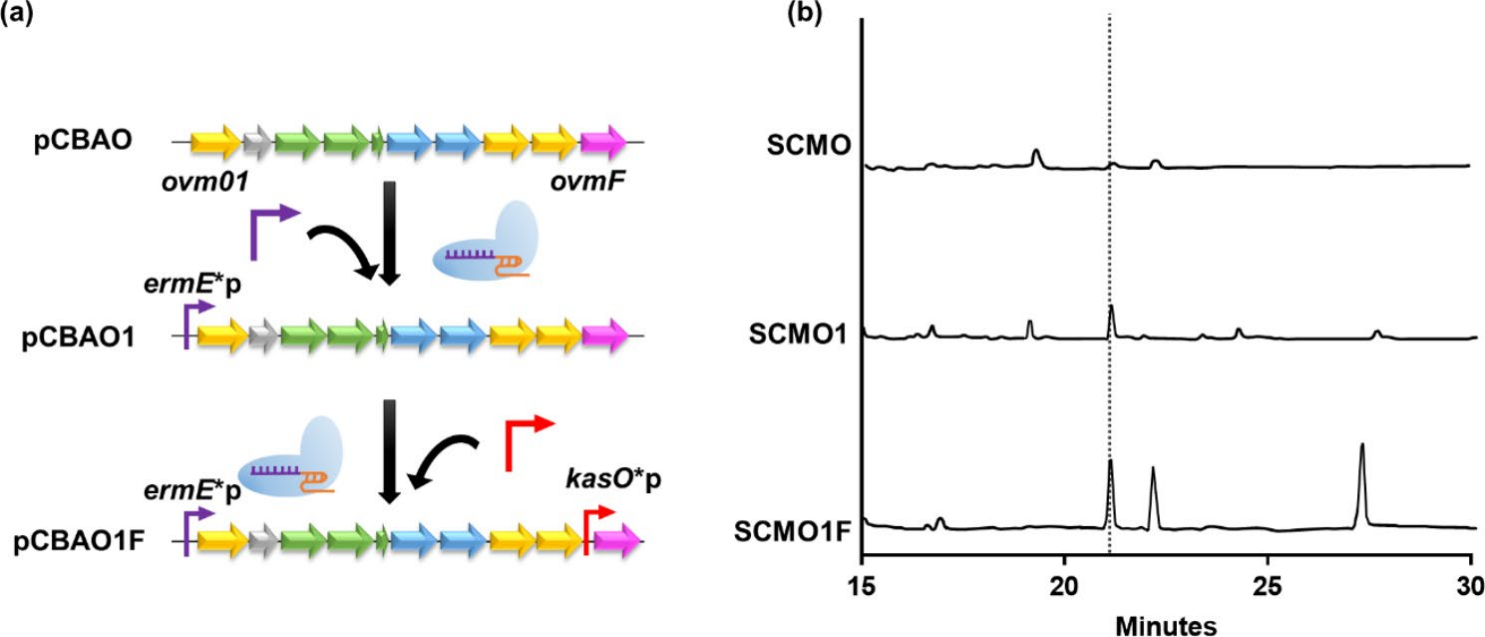
(**a**) Scheme of promoter refactoring of each BGC gene using in vitro CRISPR/Cas9. Purple: ermE*p; red: kasO*p. (**b**) LC chromatograph of oviedomycin (indicated by the dotted line)

To enhance oviedomycin production in SCMO, gene expression levels associated with *ovm* BGC were investigated. At least ten genes (*ovm01–ovmF*) are required for oviedomycin biosynthesis [34]. RT-qPCR analysis of *ovm* BGCs in SCMO revealed that *ovm01* had the lowest transcription level among all the BGC genes (Fig. S2). Thus, the *ovm01* promoter was substituted with the strong ermE* promoter known for its high activity in *S. coelicolor* [35] using in vitro CRISPR/Cas9 technology. The resultant promoter-refactored vector was named pCBAO1 (Fig. 2a), and when it was used in conjugation with *S. coelicolor* M1152, the resulting strain was named SCMO1. Oviedomycin production in SCMO1 was 12.71 mg/L, which was 10.2-fold higher than that in SCMO (Fig. 2b).

Polyketide biosynthesis often involves a crucial step where acyl carrier proteins are modified via phosphopantetheinylation by an enzyme called phosphopantetheinyl transferase (PPTase). PPTase is commonly overexpressed to enhance secondary metabolite production [36]. In the oviedomycin BGC, *ovmF* encodes a 4’-PPTase that activates the acyl carrier protein [37]. Increasing PPTase levels could enhance the production of natamycin in *Streptomyces chattanoogensis* L10 [38]. Considering this, we replaced the native promoter of *ovmF* with kasO*p, resulting in an even higher oviedomycin production compared to that observed with the SCMO1 strain. The refactored strain, SCMO1F, produced 24.96 mg/L of oviedomycin, representing a two-fold increase in production (Fig. 2b).

### Overexpression of gene manipulation targets predicted by GEM

**Fig. 3:**
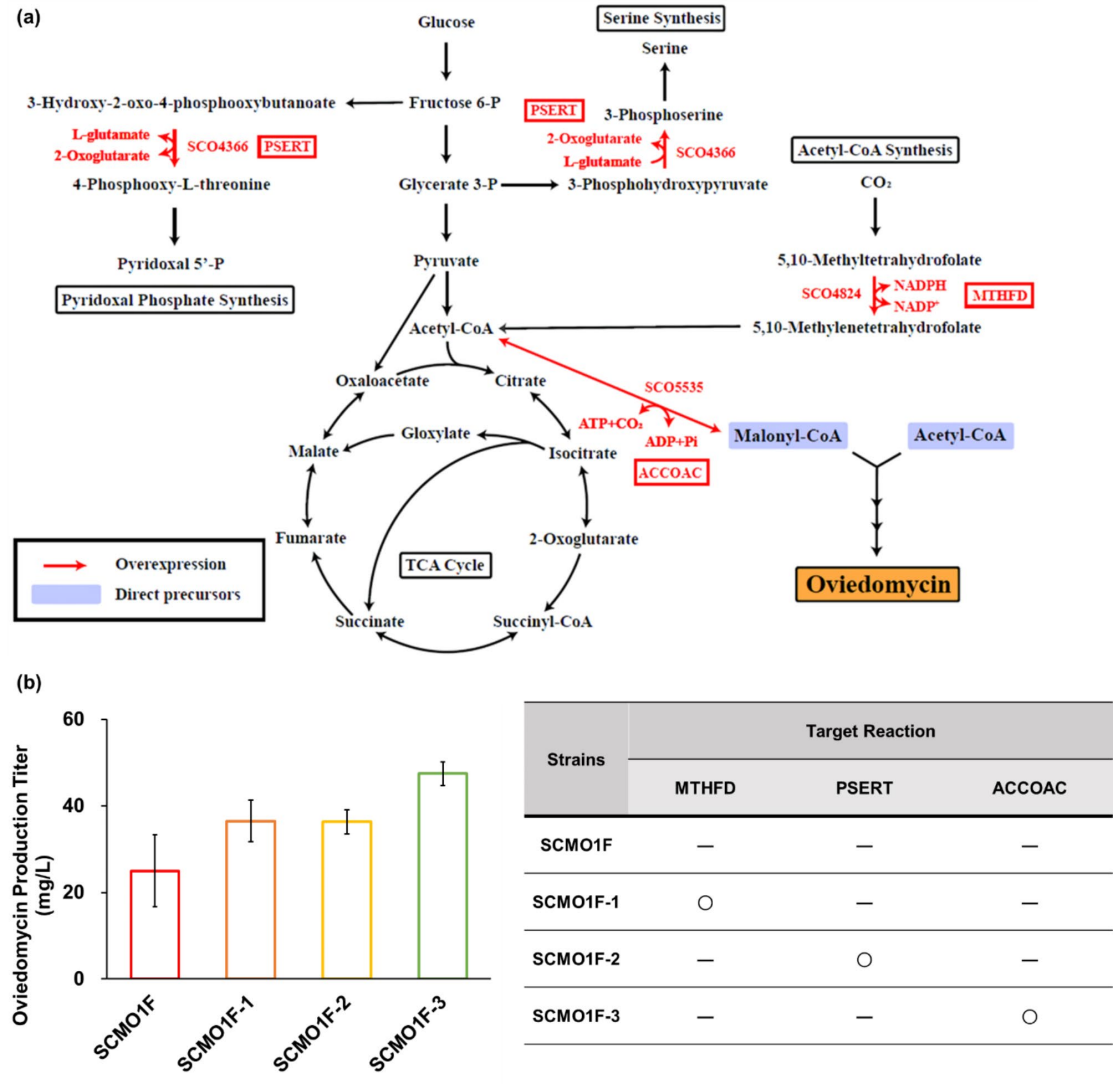
(**a**) Metabolic pathways of *S. coelicolor* M1152 that present gene manipulation targets predicted using the genome-scale metabolic model ScoSBML2119. Locus tags in red indicate overexpression targets. Reaction IDs are also presented in boxes for each gene manipulation target. Enzyme names for the presented reaction IDs are: PSERT, phosphoserine transaminase; MTHFD, methylenetetrahydrofolate dehydrogenase; and ACCOAC, acetyl-CoA carboxylase. (**b**) Analysis of oviedomycin production in SCMO1F with individual overexpression of the three target genes predicted using the GEM. In the table, ‘O’ and ‘-’ denote inclusion and absence of an overexpressed gene predicted using the GEM, respectively. Data represent the average and standard deviation of three independent experiments

We aimed to identify additional gene manipulation targets to enhance oviedomycin production. For this, we used a GEM of *S. coelicolor* with the addition of a oviedomycin biosynthetic reaction, which was named as ScoSBML2119. ScoSBML2119 was subjected to FSEOF, a gene-targeting method [39]. A total of 64 overexpression targets were initially predicted (Table S3). We eventually narrowed them down to three. The final three target reactions are mediated by phosphoserine transaminase (PSERT; encoded by SCO4366), methylenetetrahydrofolate dehydrogenase (MTHFD; encoded by SCO4824), and acetyl-CoA carboxylase (ACCOAC; encoded by SCO5535) (Fig. 3a). These reactions are directly involved in the production of malonyl-CoA or NADPH, both of which are necessary for oviedomycin production. We also made efforts to predict genes for downregulation; however, FSEOF did not provide predictions for these genes.

PSERT converts L-glutamate to 2-oxoglutarate while producing NADPH [40], and MTHFD catalyzes the 5,10-methyltetrahydrofolate dehydrogenation reaction, also leading to NADPH generation [41]. Overexpression of the corresponding genes was expected to enhance the activity of enzymes, such as oxygenases (*ovm01–03*), aromatase (*ovmA*), and ketoreductase (*ovmT*), all of which rely on NADPH as a cofactor. Additionally, the ACCOAC reaction would also be beneficial because it converts acetyl-CoA to malonyl-CoA [42], a major precursor of oviedomycin. The corresponding genes were individually overexpressed in *S. coelicolor* M1152 cells.

The target genes were inserted into an expression vector controlled by the kasO* promoter [43, 44], which was then introduced into the SCMO1F strain (Fig. S3a). This resulted in the synthesis of three new strains, SCMO1F-1, SCMO1F-2, and SCMO1F-3. The production of oviedomycin in all these engineered strains was approximately 1.46- to 1.90-fold higher compared to that observed in the SCMO1F strain (Fig. 3b). Notably, SCMO1F-3 exhibited the highest oviedomycin production titer (47.45 mg/L).

### Refactoring BGC for relieving intermediate accumulation

**Fig. 4:**
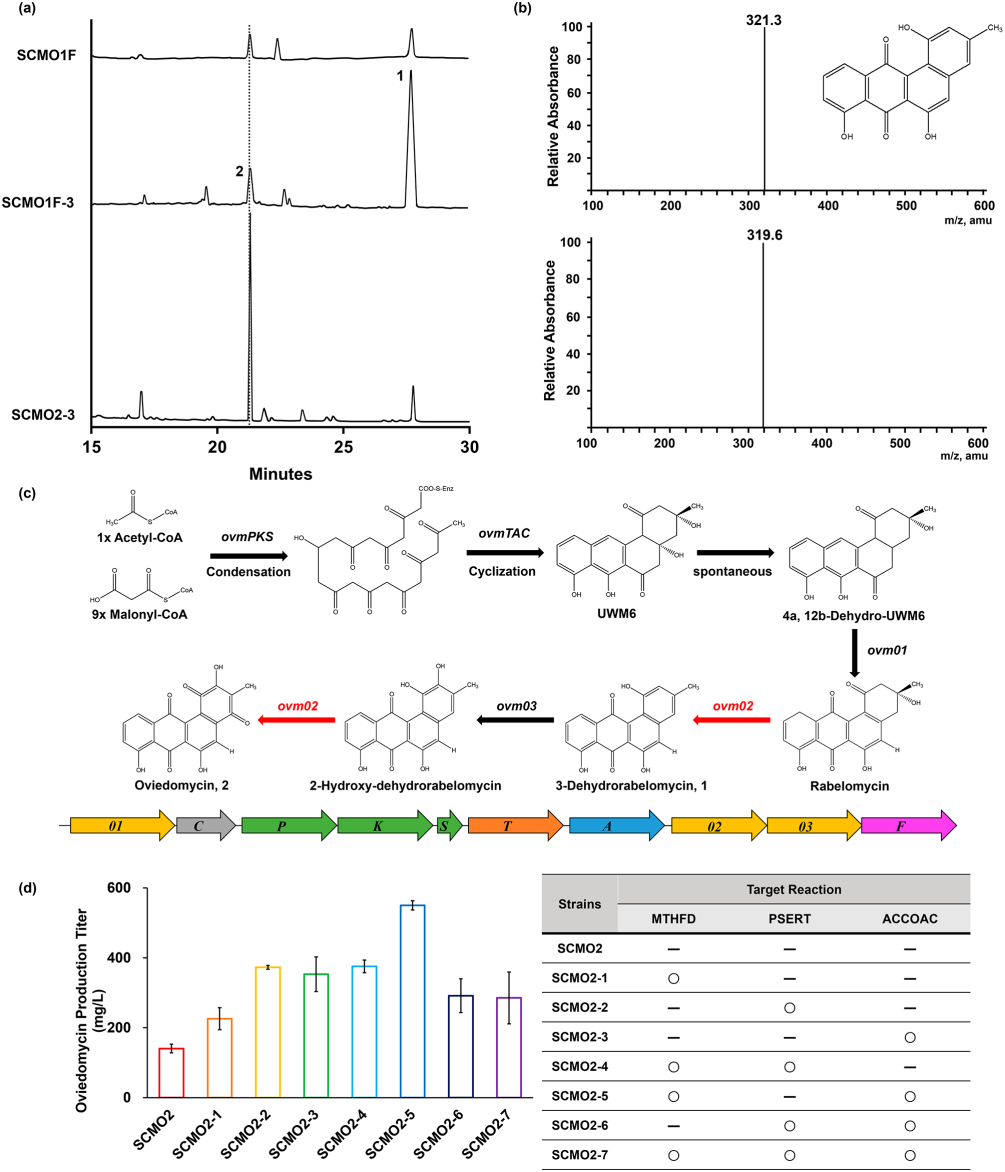
(**a**) HPLC chromatograms of cell extracts of SCMO1F, SCMO1F-3, and SCMO2-3 were analyzed. Peaks 1 and 2 are 3-dehydrorabelomycin and oviedomycin, respectively. (**b**) Results of LC/MS of 3-dehydrorabelomycin (*m/z*: 320, Up: 321.3 [*M* + H^+^]; bottom: 319.6 [*M* + H]). (**c**) Oviedomycin biosynthetic pathway. Refactoring of the target gene is indicated by the red arrow. *Ovm* BGC is shown with the corresponding pathway (yellow, oxygenase; gray, cyclase; green, polyketide synthase; orange, ketoreductase; blue, aromatase; and pink, 4’-phosphopantetheinyl transferase). (**d**) Analysis of oviedomycin production in SCMO2 with individual overexpression of the target genes, and also with overexpression of pairwise combinations of the target genes. The three target genes were predicted using the GEM. In the table, ‘O’ and ‘-’ denote the inclusion and absence of overexpressed gene(s), respectively. Data represent the average and standard deviation of three independent experiments

During the experiment focused on overexpressing the target genes using GEM to increase oviedomycin production, a new peak emerged in the HPLC chromatogram. This peak was notably prominent in samples where ACCOAC was overexpressed (Fig. 4a). Further investigation using LC-MS analysis identified this peak as 3-dehydrorabelomycin (1) in the chromatogram of SCMO1F-3 (Fig. 4a). This finding indicated the accumulation of 3-dehydrorabelomycin, an intermediate in the oviedomycin production pathway (Fig. 4b, c). We hypothesized that the increased availability of precursor molecules resulting from metabolic engineering of the host strain led to an inadequate rate of polyketide biosynthesis, causing the accumulation of intermediates. To address this issue, additional refactoring was performed to rectify the situation.

To facilitate the conversion of the intermediate metabolite 3-dehydrorabelomycin to the final product oviedomycin, we targeted *ovm02* and *ovm03*, both involved in conversion of 3-dehydrorabelomycin to oviedomycin (Fig. 4c). Given that the promoter of *ovm03* is located within *ovm02*, we hypothesized that, by refactoring the *ovm02* gene, we could simultaneously enhance the expression levels of both genes. This rationale is based on a unidirectional transcriptional mechanism comprising one operon [9].

The promoter of *ovm02* within the BGC was substituted with kasO* promoter using in vitro CRISPR/Cas9 technology, resulting in the generation of the pCBAO2 construct. The strain that was conjugated with pCBAO2 was designated as SCMO2. As a result, the quantity of 3-dehydrorabelomycin was considerably reduced, while the amount of oviedomycin was increased to 140.56 mg/L. This represents a 5.6-fold improvement compared to that observed in SCMO1F, as illustrated in Fig. 4a.

After the refactored *ovm* BGC was conjugated to *S. coelicolor* M1152, the overexpressed target genes predicted using the GEM were combined into an expression vector controlled by the kasO* promoter and then transferred into the SCMO2 strain (Fig. S3b). This led to the generation of seven different strains: SCMO2-1, SCMO2-2, SCMO2-3, SCMO2-4, SCMO2-5, SCMO2-6, and SCMO2-7. The production of oviedomycin in these strains was high, with an increase ranging from 1.6-fold to 3.9-fold than that of the SCMO2 strain (Fig. 4d).

Finally, SCMO2-5, the strain with the highest oviedomycin production, was selected for batch fermentation. The fermentation was performed using a production medium containing soybean flour (20 g/L) and mannitol (20 g/L) with antifoam and a working volume of 2 L using a 5 L bioreactor. The fermentation condition was as follows: 30 °C, agitation speed of 300 rpm, 2 vessel volume per minute (vvm), and duration of seven days [25]. As a result, 670 mg/L of oviedomycin was produced in the fermentation culture of the SCMO2-5 strain.

## Discussion

Improving the yield of drug candidates is vital to meet the growing demands of the global pharmaceutical industry [45]. Therefore, apart from discovering valuable polyketides, there is a significant need to express and enhance the production of naturally occurring bioactive compounds encoded by silent BGCs. Hence, expressing genes belonging to these silent BGCs in different host strains has garnered significant interest. However, one of the challenges in this process is finding an efficient BGC-capturing vector. To overcome this problem, we used the pCAP-BAC vector reported by Zhang et al. [32] in our study (Fig. S1). Nonetheless, the BAC vector lacked integration specificity for the host strain. As a solution, we introduced a *Streptomyces* integrated cassette to generate alternative vector (i.e., pCBA) and successfully clone the *ovm* BGC (i.e., pCBAO).

In particular, promoters play a crucial role in initiating gene expression, and optimizing them is vital for activating and improving the function of BGCs in a heterologous host strain [24]. We successfully used in vitro CRISPR/Cas9 to modify the promoters in *ovm* BGC. Changing the promoter of *ovm01*, which affects the expression of all genes within the *ovm* BGC, along with the promoter of *ovmF* gene encoding a 4’-phosphopantetheinyl transferase enzyme (PPTase), led to a successful increase in oviedomycin production. These results suggest that other BGC modifications, involving ribosome binding sites and terminators, could also enhance secondary metabolite production via targeted genetic engineering [46–48].

Systems metabolic engineering with *S. coelicolor* GEM (ScoSBML219) was employed to enhance oviedomycin production. Three specific enzymes were targeted for overexpression: MTHFD, PSERT, and ACCOAC. MTHFD and PSERT are involved in generating NADPH, whereas ACCOAC is related to malonyl-CoA production. Overexpression of MTHFS significantly increased pikromycin production in a previous study, supporting the selection of this target [25]. Similarly, ACCOAC overexpression has been utilized to boost actinorhodin production in *S. coelicolor* by providing malonyl-CoA [49]. Although the use of PSERT has not been extensively explored for enhancing the production of secondary metabolites, the results of this study revealed its positive impact among the reactions targeted using the GEM. These findings underscore the effectiveness of GEMs in identifying suitable engineering targets. A next challenge in our research is to optimize production via engineering of regulatory genes.

The metabolite profiling played a significant role in enhancing heterologous oviedomycin production. An unexpected result of ACCOAC overexpression was the observation of a peak identified as 3-dehydrorabelomycin (Fig. 4a). Increased precursor supply mediated the accumulation of this intermediate in the *ovm* pathway. Overexpression of potential rate-limiting enzymes encoded by *ovm02* and *ovm03* in the downstream pathway improved oviedomycin production. Following the refactoring process, overexpression of MTHFD and ACCOAC resulted in a highly improved oviedomycin production in the heterologous strain. These overexpressions likely rebalanced the utilization of NADPH and CoA, redirecting them towards the polyketide biosynthetic pathway. However, it is worth noting that, when SCMO2-7 co-expressed MTHFD, PSERT, and ACCOAC, oviedomycin production was decreased. This discrepancy may be attributed to the complex nature of a secondary metabolite biosynthetic pathway, which requires precise and stringent control [50–52].

By refactoring the *ovm* BGC and incorporating various engineering strategies, 670 mg/L oviedomycin was produced in a heterologous host. Similarly, Heng et al. [53] applied multiple engineering strategies for the production of armeniaspirols, a class of antibiotics, by using native *Streptomyces* producers. These results support the need of systematic engineering strategies to significantly enhance the biosynthesis of secondary metabolites that are encoded by either silent gene clusters, or those that are produced at a low production titer.

## Conclusions

Oviedomycin has been identified as a potential anticancer agent against several human cancer cell lines. However, silent BGCs in *S. antibioticus* limit the production of this compound. In this study, we aimed to activate this silent BGC to improve oviedomycin production. We developed a new BGC-capturing vector, pCBA, to safely maintain the BGC in a production host. Heterologous expression of BGC in *S. coelicolor* M1152 was confirmed, and we employed in vitro CRISPR/Cas9 and host strain engineering to increase the yield of oviedomycin. The optimizing strategy resulted in the production of 670 mg/L of oviedomycin from *S. coelicolor* M1152, which is the highest titer reported to date. Our approach provides a framework for improving the production of useful polyketides, and has the potential to accelerate advancements in the global pharmaceutical industry.

## Methods

### Conditions for culturing of bacterial strains

*E. coli* TOP10, ET12567 (pUZ8002), and various *Streptomyces* strains were subjected to standard cultivation and manipulation protocols [54, 55]. The complete list of strains and plasmids used in this study is available in Table S1, and the primer list is presented in Table S2. *S. antibioticus* NRRL 3238 was obtained from the Northern Regional Research Laboratory (USDA, Peoria, IL, USA), and *S. coelicolor* M1152 was provided by Dr. Mervyn Bibb (John Innes Centre, Norwich, UK). Cultivation of *Streptomyces* strains was performed using mannitol soya (MS) flour agar medium containing 20 g/L mannitol, 20 g/L soybean, and 20 g/L agar, which was supplemented with 10 mM MgCl2 for sporulation at 30 °C. MS broth was used for metabolite production, whereas tryptic soy broth (TSB) was used for general growth. *E. coli* strains were cultured in Luria-Bertani (LB) broth or on LB agar at 37 °C, and the appropriate antibiotics were added for selection (50 µg/mL apramycin, 50 µg/mL kanamycin, 25 µg/mL chloramphenicol, and 25 µg/mL nalidixic acid). All the chemicals and reagents were obtained from Sigma-Aldrich.

### Construction of a BGC cloning vector

The genomic DNA of *S. antibioticus* (accession number KY129858) was extracted using a Promega Genomic DNA Purification Kit (Promega Corp. Madison, WI, USA) according to the manufacturer’s protocol. Target BGCs were amplified from *S. antibioticus* NRRL 3238 genomic DNA using PrimeSTAR GXL DNA polymerase (TaKaRa, Kusatsu, Japan). The pCAP-BAC plasmid, obtained from Addgene (#120,229), was used for BGC cloning. To construct an *E. coli*-*Streptomyces* conjugated shuttle vector, the apramycin resistance gene, phage φC31 integrase, and traJ from pSET152 were cloned into pCAP-BAC, resulting in the formation of pCAP-BAC-Apr (pCBA). Primer synthesis and DNA sequencing were conducted by BIONICS and Macrogen, respectively. Cloning was performed using a ligation mixture and Gibson assembly master mixture obtained from New England Biolabs (NEB, UK).

### In vitro CRISPR/Cas9-based refactoring of the cloning vector

The cloning vector (pCBAO) for BGC was engineered using the CRISPR/Cas9 technology to modify the gene promoter of the target BGC. Cas9 protein was obtained from NEB, and sgRNAs were synthesized at Macrogen. To design sgRNAs, a gene sequence targeted for editing was analyzed to identify a 20 bp guide sequence along with a protospacer adjacent motif (PAM) sequence located near the start codon. The CRISPy-web program [56] was used to design target sites for sgRNAs. The cloning vector for BGC was concentrated using a freeze dryer (HyperVac 2200, LaboGene).

An in vitro Cas9-mediated editing was performed with slight modifications [57]. For the in vitro Cas9 editing, a reaction mixture of 20 µL was prepared, containing a 500 ng of Cas9 protein, 250 ng of sgRNA, and NEB buffer 3.1. The mixture was then incubated at 25 °C for 10 min. Subsequently, 2 µL of the plasmid (2 µg) was mixed with the Cas9-sgRNA reagent and incubated at 37 °C for 1 h. To terminate the reaction, 2 µL of a stop solution containing 30% glycerol, 1.2% SDS, and 250 mM EDTA pH 8.0 was added, followed by an additional incubation at 37 °C for 15 min. Next, the reaction mixture was treated with 2 µL of 4 mg/mL RNase (Promega Corp. Madison, WI, USA) at 37 °C for 15 min. Finally, the Cas9-digested DNA was recovered using ethanol precipitation [58]. Promoter cassettes for each target gene were synthesized using the vectors pCAP03-ermE*p (Addgene #12,034) and pSET152-kasO*p from Cho et al. [25]. The Cas9-digested DNA and promoter cassettes were ligated using the Gibson assembly.

### Heterologous expression, fermentation, and high-performance liquid chromatography (HPLC) analysis

pCBAO and its derivatives were transferred to *E. coli* ET12567 (pUZ8002) via electroporation and then conjugated to *S. coelicolor* M1152. The exoconjugants were cultured in the MS medium supplemented with 10 mM MgCl2.

After incubating at 30 °C for 16 h, the plates were covered with 20 µL of nalidixic acid and 25 µL of apramycin [59]. Cultures were grown until the exoconjugants appeared. To produce oviedomycin, a spore suspension of *S. coelicolor* M1152 was used to inoculate the tryptic soy broth medium, followed by incubation for 48 h. A 5% seed culture inoculum was used to inoculate the fermentation medium (MS medium). Fermentation was performed at 30 °C and 250 rpm in a rotary incubator for seven days. After fermentation, 50 mL of liquid culture broth was mixed with an equal volume of ethyl acetate and sonicated for 20 min. The resulting organic phase was transferred and subsequently evaporated under conditions involving a vacuum at a low temperature. The dried metabolites were dissolved in ethanol and filtered using a 0.22 μm membrane filter to eliminate particles before subjecting the samples to HPLC [8].

HPLC was conducted using a Waters Alliance 2695 Separation Module HPLC system. Metabolite analysis was performed on an Eclipse XDB C18 column (particle size: 5 μm, 4.6 × 250 mm) with a flow rate of 1 mL/min and a photo diode array (PDA) detector. The elution process followed a 40-min gradient method with the eluent initially consisting of 5% of solvent B at time T = 0. After 30 min, the eluent comprised 100% of solvent B, and an isocratic gradient with 100% solvent B was maintained from T = 33 to T = 34 min. Finally, an isocratic gradient was maintained with 5% of solvent B from T = 40 min onward. Eluent A comprised distilled water with 0.1% (v/v) trifluoroacetic acid, and Eluent B was acetonitrile. The column temperature was maintained at 40 °C, and the injection volume was 10 µL.

Liquid chromatography-tandem mass spectrometry (LC-MS/MS) was performed using an Agilent HPLC 1200 series triple-quadrupole mass spectrometry system. Metabolites were analyzed on a SunFire C18 column (particle size: 3.5 μm, 2.1 × 100 mm). Mass spectrometry-electrospray ionization (MS-ESI) spectra were obtained in positive and negative ion modes. The ionization parameters are as follows: ±4.5 kV ionization voltage, 20 psi nebulizer gas, 50 psi heater gas, and 500 °C source temperature. The mass range for the analysis was set between 200 and 600 *m/z*. HPLC-prep analysis was performed using a ThermoDionex Ultimate3000 preparative HPLC system. Metabolite analysis was performed on an Agilent Prep-C18 column (particle size: 10 μm, 250 × 10 mm). The analytical conditions were the same as above except for a flow rate of 5 mL/min and an injection volume of 50 µL. Preparative oviedomycin was used as an authentic standard for comparison.

### Reverse transcription quantitative PCR (RT-qPCR) of engineered strains

For the cultivation of SCMO and SCMO1 strains to produce oviedomycin, the methods described for oviedomycin production were followed. After three days of growth in the production media, 1 mL of culture sample was collected and centrifuged to form a pellet, which was subsequently cleaned using RNase-free water. RNA was extracted using a Zymo Research RNA extraction kit specified for gram-positive bacterial cells. Using the SuperScript III Reverse Transcriptase kit (Invitrogen), the extracted RNA was converted to cDNA.

RT-qPCR was performed using SYBR Green on an Agilent Real-time PCR System. The relative expression level of the target genes was normalized by using *hrdB* gene as an endogenous control.

### Genome-scale metabolic simulation for predicting gene manipulation targets

A high-quality *S. coelicolor* GEM iKS1317 [60] was used to predict gene manipulation targets. To simulate an oviedomycin-producing strain, a new oviedomycin biosynthetic reaction (reaction ID: ‘OVM_SYN’) was incorporated into iKS1317. The resulting GEM, ScoSBML2119, is presented as Supplementary Data in the Systems Biology Markup Language (SBML). Subsequently, ScoSBML2119 was subjected to a gene-targeting method called flux scanning based on enforced objective flux (FSEOF) to identify gene manipulation targets for increasing oviedomycin production [61]. FSEOF was performed with the objective of maximizing cell growth, while gradually increasing the oviedomycin production rate from an initial flux value to a theoretical maximum formation rate value. As a result, reactions that showed a positive correlation with the oviedomycin production rate were considered overexpression targets. For the FSEOF implementation, constraints were applied to the exchange reaction fluxes of nutrients. The lower bounds of all exchange reactions, except for glucose, were set to − 1000 mmol/g DCW/h. The glucose exchange reaction was constrained to − 0.8 mmol/g DCW/h. The GEM was edited by using COBRApy [62], and FSEOF was implemented by using Cameo [39].

### Electronic supplementary material

Below is the link to the electronic supplementary material.

Supplementary Material 1

## Data Availability

All data generated or analyzed in this study are included in this article and its additional file.

## Abbreviations

ACCOAC: Acetyl-CoA carboxylase
BAC: Bacterial artificial chromosome
BGC: Biosynthetic gene cluster
Cas9: CRISPR-associated protein 9
CRISPR: Clustered regularly interspaced short palindromic repeats
EDTA: Ethylenediaminetetraacetic acid
FSEOF: Flux scanning based on enforced objective flux
GEM: Genome-scale metabolic model
HPLC: High-performance liquid chromatography
LB: Luria-Bertani
LC-MS: Liquid chromatography-mass spectrometry
LC-MS/MS: Liquid chromatography-tandem mass spectrometry
MS: Mannitol soya
MS-ESI: Mass spectrometry-electrospray ionization
MTHFD: Methylenetetrahydrofolate dehydrogenase
ovm: Oviedomycin
PAM: Protospacer adjacent motif
PPTase: Phosphopantetheinyl transferase
PSERT: Phosphoserine transaminase
SBML: Systems Biology Markup Language
SDS: Sodium dodecyl sulfate
sgRNA: Single guide RNA
TSB: Tryptic soy broth
UTR: Untranslated region
vvm: Vessel volume per minute
