# Supplementary material for: Heterologous overproduction of oviedomycin by refactoring biosynthetic gene cluster and metabolic engineering of host strain Streptomyces coelicolor
Source: Microb Cell Fact. 2023 Oct 14;22:212. doi: 10.1186/s12934-023-02218-8 (PMC10576301; doi:10.1186/s12934-023-02218-8)
Supplement: Supplementary file 1 — Supplementary Material 1 [file 12934_2023_2218_MOESM1_ESM.docx]

Supplementary Information

Heterologous overproduction of oviedomycin by refactoring biosynthetic gene cluster and metabolic engineering of host strain *Streptomyces coelicolor*

Boncheol Gu^1^, Duck Gyun Kim^1^, Do-Kyung Kim^1^, **Minji Kim^2^**, Hyun Uk Kim^2^, Min-Kyu Oh^1^

^1^Department of Chemical & Biological Engineering, Korea University, Seoul 02841, Republic of Korea

^2^Department of Chemical and Biomolecular Engineering (BK21 four), Korea Advanced Institute of Science and Technology (KAIST), Daejeon, 34141, Republic of Korea

Table. S1 Strains and plasmids in this study.

| Strains | Genotype or Description | References |
| --- | --- | --- |
| *Escherichia coli* |  |  |
| TOP10 | A derivative of *E. coli* K12 strain. *F*_ *mcr*A Δ(*mrr-hsdRMS-mcrBC*) φ80*lacZ*Δ*M15* Δ*lacX*74 *endA*1 *galE*15 *galK*16 *rpsL* (*StrR*) *nup*G *recA*1 *araD*139 Δ(*ara-leu*)7697 | Invitrogen |
| ET12567(pUZ8002) | A methylation-deficient strain for conjugation-based gene transfer; carrying pUZ8002 plasmid | (1) |
| *Streptomyces coelicolor* M1152 | A derivative *Streptomyce coelicolor* A3(2) strain. ∆*act* ∆*red* ∆*cpk* ∆*cda* *rpoB* (C1298T) | (2) |
| SCMO | M1152 harboring pCBAO | This study |
| SCMO1 | M1152 harboring pCBAO1 | This study |
| SCMO1F | M1152 harboring pCBAO1F | This study |
| SCMO2 | M1152 harboring pCBAO2 | This study |
| Plasmids |  |  |
| pCAP-BAC | Low copy maintenance of large constructs in *E. coli* | (3) |
| pSET152-*kasO**p | An integrated expression plasmid of *Streptomyces* with *kasO**p | (4) |
| pCBA | Integrated phage φC31 integrase, apramycin^R,^ tra, from pSET152 and ori2, repE. sopA, sopB, sopC from pCAP-BAC | This study |
| pCBAO | Cloned *ovm* BGC into pCBA | This study |
| pCBAO1 | Refactoring ovm01 followed by *ermE**p | This study |
| pCBAO1F | Refactoring ovm01 with *ermE**p and ovmF with *kasO**p | This study |
| pCBAO1F-1 | Insert a *MTFHD* with *kasO**p into pCBAO1F | This study |
| pCBAO1F-2 | Insert a *PSERT* with *kasO**p into pCBAO1F | This study |
| pCBAO1F-3 | Insert a ACCOAC with *kasO**p into pCBAO1F | This study |
| pCBAO2 | Refactoring ovm01 with *ermE**p, ovmF and ovm02 with *kasO**p | This study |
| pCBAO2-1 | Insert a *MTFHD* with *kasO**p into pCBAO2 | This study |
| pCBAO2-2 | Insert a *PSERT* with *kasO**p into pCBAO2 | This study |
| pCBAO2-3 | Insert a ACCOAC with *kasO**p into pCBAO2 | This study |
| pCBAO2-4 | Insert *MTFHD* and *PSERT* with *kasO**p into pCBAO2 | This study |
| pCBAO2-5 | Insert *MTFHD* and *ACCOAC* with *kasO**p into pCBAO2 | This study |
| pCBAO2-6 | Insert *PSERT* and *ACCOAC* with *kasO**p into pCBAO2 | This study |
| pCBAO2-7 | Insert *MTFHD*, *PSERT* and *ACCOAC* with *kasO**p into pCBAO2 | This study |

Table. S2 Primers used in this study.

| Name | 5’ to 3’ sequence |
| --- | --- |
| pSET152_F | GAAGATCCTTTGATCTTTTCTACGGGG |
| pSET152_R | CAGGCTTCCCGGGTGTCT |
| ovm_BGC_F | **AGTGCCACCTGGGTCCTTTTCATCACGTGCTATAAAAATAATTATAATTT**CGACATGACGTGCCCTCAG |
| ovm_BGC_R | **CTGGTGCTTCCTCTCACTATAGGGCGAATTGGAGCTCCACCGCGGTGGC**TCATGGGTACCCTCCGGTTG |
| MTHFD_F | ATATAT*CATATG*ACCGCCCAGATTCTCGATGG |
| MTHFD_R | ATATAT*GGATCC*TCAGCCGGCACTGCGCTC |
| ACCOAC_F | ATATAT*CATATG*ACCGTTTTGGATGAGGCGC |
| ACCOAC_R | ATATAT*GCGGCCGC*TCACTGCGGCGGGTTGCC |
| PSERT_F | ATATAT*CATATG*AAGCCCGCGGACGGT |
| PSERT_R | ATATAT*GGATCC*TCACAGCTTCTCGATGACGTGGTC |
| sgRNA |  |
| ovm01 | ACGCAACAGGAAAGGAGGCG |
| ovmF | GAAACGCTCCCGGTGCCGGA |
| ovm02 | CGAAGCACGGCGGAACCATG |
| Rescue DNA |  |
| *ermE**p_ovm01_F | **CGGGACGTACGCAACAGGAA**tcgatcttgacggctggcgagaggtgcggggaggatctgaccgacgcggtccacacgtggcaccgcgatgctgttgtgggca |
| *ermE**p_ovm01_R | **TCGACGAGGGCAGCGTCCAT**atggggacctcctggggtgcgttggaccgctggatcctaccaaccggcacgattgtgcccacaacagcatcgcgg |
| *kasO**p_ovmF_F | **CGGTACCGGAAACGCTCCCG**tgttcacattcgaacggtctctgctttgacaacatgctgtgcggtgttgtaaagtcgtggccagg |
| *kasO**p_ovmF_R | **ACAGGTCCACGCGCCGGCCGTCCGGCACATGCGCA**ctcccccagtcctgcacgctgtcgtattctcctggccacgactttacaac |
| *kasO**p_ovm02_F | **AGGACTACGCCGAAGCACGGCGGAACCATGCGGACTGA**tgttcacattcgaacggtctctgctttgacaacatgctgtgcggtgttgtaaagtcgtgg |
| *kasO**p_ovm02_R | **GCCCGCGCCGACGACGAGGACGTCAGTCCGCATATGCGCA**ctcccccagtcctgcacgctgtcgtattctcctggccacgactttacaac |
| RT-qPCR |  |
| ovm01_F | CGTCAACCTGGGCTGGAAGC |
| ovm01_R | GCATCTCGCTGCCGCTGA |
| ovmC_F | TGAAGCACAGCACGTTGATC |
| ovmC_R | CGGTACTTGAACAGCTGACG |
| ovmP_F | AAGAACTTCTGGAGCCTGCT |
| ovmP_R | TGTTCCTCCGGATCGAAGTC |
| ovmK_F | CATCGAGGAGGGGCTGATC |
| ovmK_R | CAGGTCCAGGTCGTAGCC |
| ovmS_F | GTCGACGACCTCAAGCGC |
| ovmS_R | GAGGAAGTCGGCGCTGTC |
| ovmT_F | GACACGAACCTCAACAGCG |
| ovmT_R | GAGGCGATGTTGATGATCCG |
| ovmA_F | GGAAGATCGCCTACAAGCAG |
| ovmA_R | CGTGATGTTGTCGGTGTTGA |
| ovm02_F | CCTCAACCTCGGTCTCCAG |
| ovm02_R | CTCGGAGTGGTACGTGTCC |
| ovm03_F | CGGAAACTGAGCAACCTGAC |
| ovm03_R | TGATCAGCACGTACGGGTAG |
| ovmF_F | CTCACCTCACCTCACCTCAC |
| ovmF_R | CTCCGGTTGCCTTGCCAA |

In primer sequences, italic, bold, and small characters represent enzyme sites, homologous region to genomic DNA, and promoter region, repectively.)

Table. S3 Overexpression targets obtained from GEM analysis.

| Targets | Name | Locus ID |
| --- | --- | --- |
| ACCOAC | Acetyl-CoA carboxylase | (SCO2445 or (SCO5535 and SCO5536)) and (SCO2777 or SCO4921 or SCO6271) |
| ACLS | Acetolactate synthase | (SCO5512 or SCO2769 or SCO6584) and SCO5513 |
| ADK1 | Adenylate kinase | SCO4723 |
| ADSK | Adenylyl-sulfate kinase | SCO6099 |
| ADSL1r | Adenylsuccinate lyase | SCO1254 |
| ADSL2r | Adenylosuccinate lyase | SCO1254 |
| ADSS | Adenylosuccinate synthase | SCO3629 |
| AHSERL2 | O acetylhomoserine thiol lyase | SCO1294 or SCO4958 |
| AICART | Phosphoribosylaminoimidazolecarboxamide formyltransferase | SCO4814 |
| AIRCr | Phosphoribosylaminoimidazole carboxylase | SCO3059 and SCO3060 |
| ASP1DC | Aspartate 1-decarboxylase | SCO0978 or SCO3416 |
| ASPTA | Aspartate transaminase | SCO4645 or SCO3658 |
| BPNT | 3',5'-bisphosphate nucleotidase | SCO5161 |
| CYSTS | Cystathionine beta synthase | SCO3077 |
| CYTK1 | Cytidylate kinase (CMP) | SCO1760 |
| DHAD1 | Dihydroxy-acid dehydratase (2,3-dihydroxy-3-methylbutanoate) | SCO1176 or SCO1888 or SCO3345 |
| DPCOAK | Dephospho-CoA kinase | SCO1996 |
| DPR | 2-dehydropantoate 2-reductase | SCO0462 or SCO6562 |
| FBA3 | Sedoheptulose 1,7-bisphosphate D-glyceraldehyde-3-phosphate-lyase | SCO3649 or SCO5852 |
| G6PDH1b | beta-D-glucose-6-phosphate:NAD+ 1-oxidoreductase | SCO1937 or SCO6661 |
| G6PDH2r | Glucose 6-phosphate dehydrogenase | SCO1937 or SCO6661 |
| G6PI | Glucose 6 phosphate isomerase | SCO1942 or SCO6659 |
| GAPD | Glyceraldehyde-3-phosphate dehydrogenase | SCO1947 or SCO7040 or SCO7511 |
| GARFT | Phosphoribosylglycinamide formyltransferase | SCO4813 |
| GHMT2r | Glycine hydroxymethyltransferase, reversible | SCO4837 or SCO5470 or SCO5364 |
| GLNS | Glutamine synthetase | SCO1613 or SCO2198 or SCO2210 or SCO2241 or SCO6962 |
| GLUPRT | Glutamine phosphoribosyldiphosphate amidotransferase | SCO4086 |
| GLYCL | Glycine Cleavage System | SCO5471 and SCO1378 and SCO5472 and (SCO0884 or SCO2180 or SCO4919) |
| H2Ot | H2O transport via diffusion | s0001 |
| IMPC | IMP cyclohydrolase | SCO4814 |
| KARA1i | acetohydroxy acid isomeroreductase | SCO5514 or SCO7154 |
| METB1 | Metb1 (rev) | SCO1294 or SCO4958 |
| MOHMT | 3-methyl-2-oxobutanoate hydroxymethyltransferase | SCO2256 |
| MTHFC | Methenyltetrahydrofolate cyclohydrolase | SCO4824 |
| MTHFD | Methylenetetrahydrofolate dehydrogenase (NADP) | SCO4824 |
| NDPK1 | Nucleoside-diphosphate kinase (ATP:GDP) | SCO2612 |
| NDPK3 | Nucleoside-diphosphate kinase (ATP:CDP) | SCO2612 |
| NH4t | Ammonia reversible transport | SCO5583 or s0001 |
| PANTS | Pantothenate synthase | SCO3383 |
| PAPSR | Phosphoadenylyl-sulfate reductase (thioredoxin) | SCO6100 and (SCO0885 or SCO3889 or SCO5419 or SCO5438) |
| PFK_3 | Phosphofructokinase (s7p) | SCO5426 or SCO2119 or SCO1214 |
| PGCD | Phosphoglycerate dehydrogenase | SCO3478 or SCO5515 |
| PGIA | Glucose-6-phosphate isomerase (g6p-A) | SCO1942 or SCO6659 |
| PGK | Phosphoglycerate kinase | SCO1946 |
| PIt2r | Phosphate reversible transport via symport | SCO4138 or SCO1845 |
| PNTK | Pantothenate kinase | SCO3380 or SCO4738 |
| PPA_1 | Pyrophosphate energized proton pump | SCO3409 or SCO3547 |
| PPCDC | Phosphopantothenoylcysteine decarboxylase | SCO1477 |
| PRAGSr | Phosphoribosylglycinamide synthase | SCO4068 |
| PRAIS | Phosphoribosylaminoimidazole synthase | SCO4087 |
| PRASCSi | Phosphoribosylaminoimidazolesuccinocarboxamide synthase | SCO4071 |
| PRFGS | Phosphoribosylformylglycinamidine synthase | SCO4077 and SCO4078 and SCO4079 |
| PRPPS | Phosphoribosylpyrophosphate synthetase | SCO0782 or SCO3123 |
| PSERT | Phosphoserine transaminase | SCO4366 |
| PSP_L | Phosphoserine phosphatase (L-serine) | SCO1808 or SCO3691 |
| PTPATi | Pantetheine-phosphate adenylyltransferase | SCO5568 |
| RPE | Ribulose 5-phosphate 3-epimerase | SCO1464 |
| RPI | Ribose-5-phosphate isomerase | SCO0579 or SCO1224 or SCO2627 |
| SADT | Sulfate adenylyltransferase | SCO6097 and SCO6098 |
| SO4t2 | Sulfate transport in via proton symport | SCO3276 |
| TALAb | sedoheptulose-7-phosphate:D-glyceraldehyde-3-phosphate glyceronetransferase | SCO1936 or SCO6662 |
| TKT1 | Transketolase | SCO1935 or SCO6497 or SCO6663 |
| TPI | Triose-phosphate isomerase | SCO0578 or SCO1945 |
| TRDR | Thioredoxin reductase (NADPH) | (SCO3890 or SCO6834 or SCO7298) and (SCO0885 or SCO3889 or SCO5419 or SCO5438) |


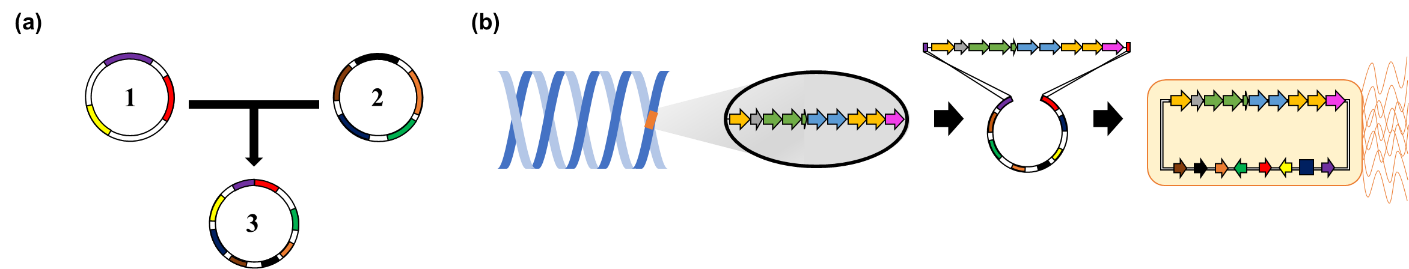
Fig. S1 (a) To capture a BGC, we combined two plasmids; pSET152 (1) and pCAP-BAC (2) to make a pCAP-BAC-Apr (pCBA) (3), Purple : phage φC31 integrase, Red : AparamycinR, Yellow : tra from pSET152, Navy : ori2 , Brown : repE, Black : sopA, Orange : sopB, Green : sopC from pCAP-BAC. (b) gDNA was extracted from *Streptomyces antibioticus* NRRL 3238, and then *ovm* BGC was synthesized by PCR method. *Ovm* BGC PCR product, included homology region (50 bp) of pCBA, was ligated into linear pCBA. *Ovm* BGC captured plasmid (pCBAO) was transformed into *E. coli* ET12567(pUZ8002).


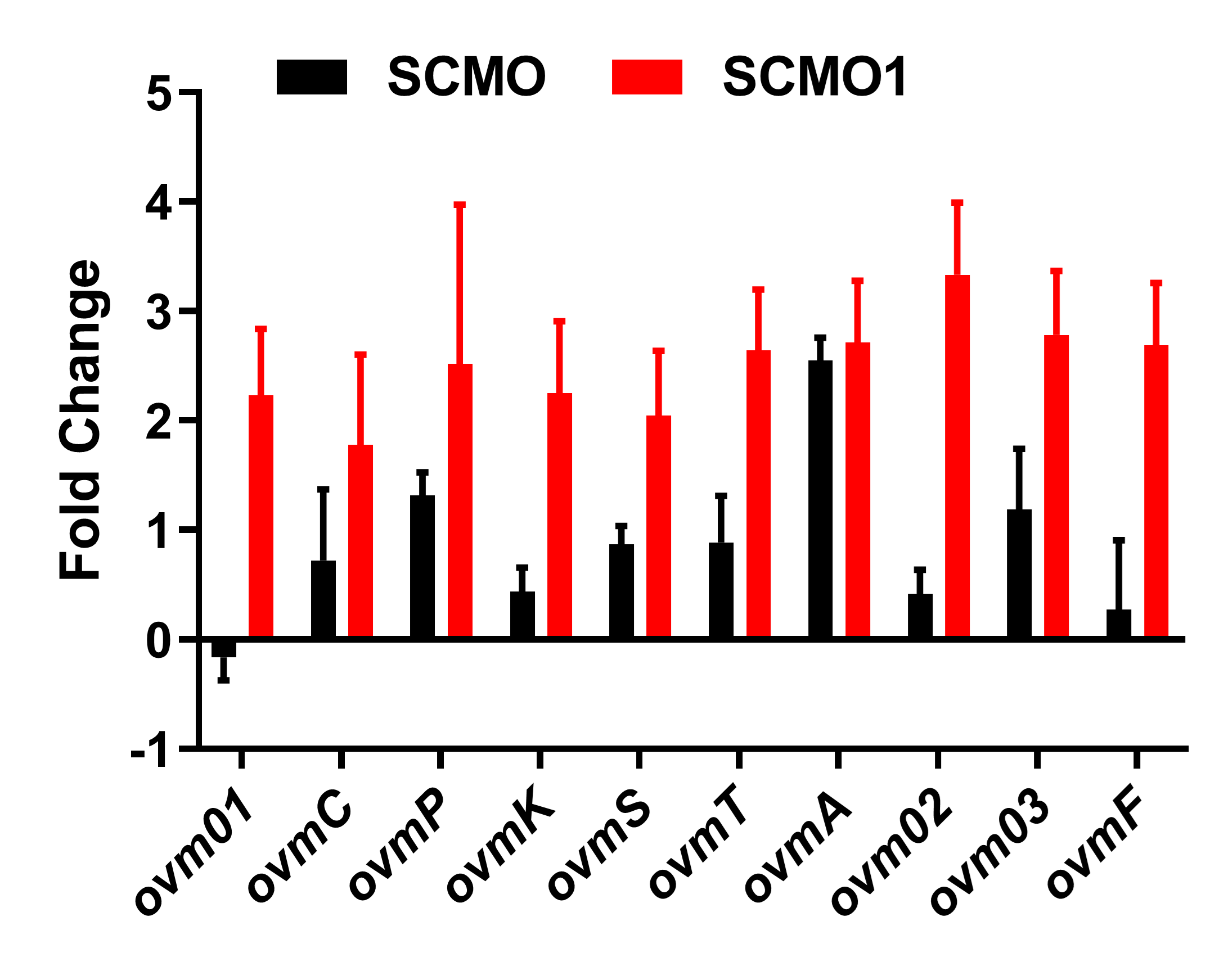


Fig. S2 RT-qPCR analysis on the fold change of SCMO and refactoring strains SCMO1. Relative expression was normalized by housekeeping gene, *hrdB*. These data represented the averages of three independent experiments ± standard deviations.


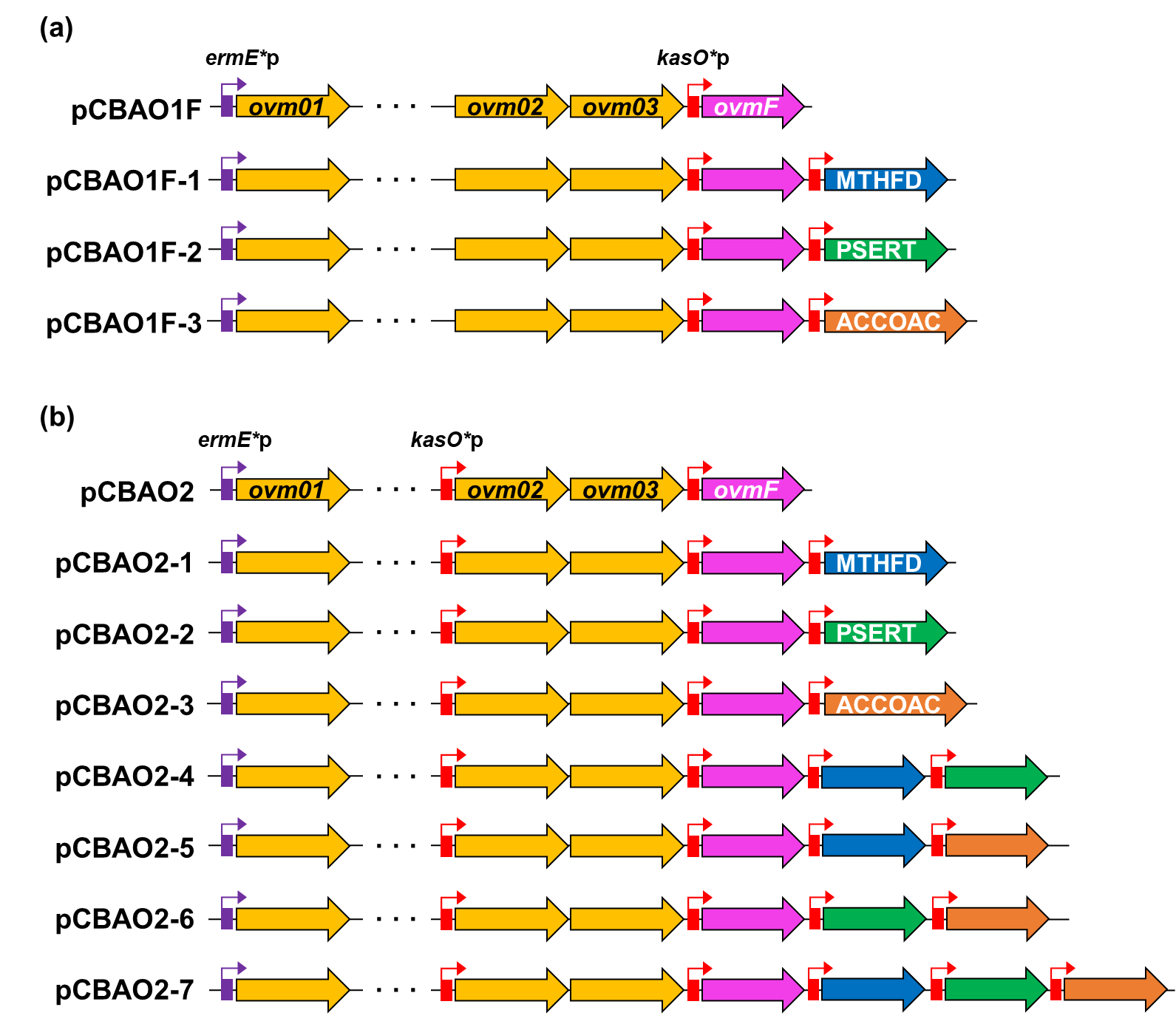


Fig. S3 Plasmid construction for overexpressing three candidate genes from GEM into pCBAO1F (a) and pCBAO2 (b)

Reference

1. Macneil DJ, Gewain KM, Ruby CL, Dezeny G, Gibbons PH, Macneil T. Analysis of *Streptomyces avermitilis* genes required for avermectin biosynthesis utilizing a novel integration vector. Gene 1992,111:61-68. https://doi.org/10.1016/0378-1119(92)90603-m.

2. Gomez‐Escribano JP, Bibb MJ. Engineering *Streptomyces coelicolor* for heterologous expression of secondary metabolite gene clusters. Microbial biotechnology 2011, 4:207-215. https://doi.org/10.1111/j.1751-7915.2010.00219.x.

3. Bauman KD, Li J, Murata K, Mantovani SM, Dahesh S, Nizet V, Luhavaya H, Moore BS. Refactoring the Cryptic Streptophenazine Biosynthetic Gene Cluster Unites Phenazine, Polyketide, and Nonribosomal Peptide Biochemistry. Cell Chem Bio 2019, 26:724-736.e7. https://doi.org/10.1016/j.chembiol.2019.02.004.

4. Cho MK, Lee BT, Kim HU, Oh MK. Systems metabolic engineering of *Streptomyces venezuelae* for the enhanced production of pikromycin. Biotechnology and Bioengineering 2022. https://doi.org/10.1002/bit.28114.
